# Can sexual selection theory inform genetic management of captive populations? A review

**DOI:** 10.1111/eva.12229

**Published:** 2014-10-29

**Authors:** Rémi Chargé, Céline Teplitsky, Gabriele Sorci, Matthew Low

**Affiliations:** 1 Department of Biological and Environmental Science, Centre of Excellence in Biological Interactions, University of JyväskyläJyväskylä, Finland; 2 Centre d'Ecologie et de Sciences de la Conservation UMR 7204 CNRS/MNHN/UPMC, Muséum National d'Histoire NaturelleParis, France; 3 Biogéosciences, UMR CNRS 6282, Université de BourgogneDijon, France; 4 Department of Ecology, Swedish University of Agricultural SciencesUppsala, Sweden

**Keywords:** conservation biology, evolutionary theory, sexual selection

## Abstract

Captive breeding for conservation purposes presents a serious practical challenge because several conflicting genetic processes (i.e., inbreeding depression, random genetic drift and genetic adaptation to captivity) need to be managed in concert to maximize captive population persistence and reintroduction success probability. Because current genetic management is often only partly successful in achieving these goals, it has been suggested that management insights may be found in sexual selection theory (in particular, female mate choice). We review the theoretical and empirical literature and consider how female mate choice might influence captive breeding in the context of current genetic guidelines for different sexual selection theories (i.e., direct benefits, good genes, compatible genes, sexy sons). We show that while mate choice shows promise as a tool in captive breeding under certain conditions, for most species, there is currently too little theoretical and empirical evidence to provide any clear guidelines that would guarantee positive fitness outcomes and avoid conflicts with other genetic goals. The application of female mate choice to captive breeding is in its infancy and requires a goal-oriented framework based on the needs of captive species management, so researchers can make honest assessments of the costs and benefits of such an approach, using simulations, model species and captive animal data.

## Introduction

Because of increasingly imperiled wildlife habitats (Pimm et al. 1995; Barnosky et al. 2011), wildlife conservation managers often incorporate *ex-situ* conservation policies to mitigate species loss (e.g., captive breeding programs). In these programs, species may be ‘preserved’ in captivity awaiting release at an unspecified future date or captive breeding used in a supportive role to supplement dwindling wild populations (Fa et al. 2011). Reintroductions (or supplementations) from captive populations have increased exponentially in recent years and are a valuable tool in many species conservation programs (Allendorf and Luikart 2007; Ewen 2012) and commercial systems (Laikre et al. 2010; Neff et al. 2011). However, there is compelling evidence that captivity-induced genetic changes of these populations contribute to reduce rates of reintroduction/supplementation success (Ford 2002; Woodworth et al. 2002; Milot et al. 2013).

Because the main goal of supportive breeding is to release individuals that not only reinforce the population in terms of its size but also its evolutionary potential, captive breeding and release strategies must consider the dual issue of quantity and quality of the individuals released (Fa et al. 2011; Neff et al. 2011). Enough individuals need to be released to overcome small-population limiting factors (e.g., environmental and demographic stochasticity, Allee effects), in addition to being well adapted to their environment and able to respond to future selection pressures. Thus, for these reintroduced individuals to have a good chance at positively impacting on the population (or succeeding in establishing), the potential negative genetic consequences of captive breeding should be minimized: that is, inbreeding depression, the loss of genetic diversity, and genetic adaptation to captivity (Lacy 1994; Ballou and Lacy 1995; Frankham 2008).

Inbreeding and random genetic drift are consequences of small populations, like those in captive breeding programs or endangered wild populations (Allendorf and Luikart 2007). Inbreeding arises because mating among relatives is more likely in small populations, and this allows the expression of recessive deleterious alleles (Charlesworth and Willis 2009). Genetic drift is the main process by which captive populations lose genetic variation (Lacy 1987), and occurs because allele frequencies randomly fluctuate between generations, with the increasing potential for some alleles being lost completely in small populations through this random process. Thus, the fitness consequences can be dramatic if it means the loss of beneficial alleles or the fixation of deleterious mutations. Captive populations face an additional genetic risk because selection on traits vital for survival in the wild is relaxed: there are no predators, diseases are treated, food is provided *ad libitum* and mate choice is often circumvented. Rare alleles that are deleterious in the wild may thus become more frequent in captive populations (Laikre 1999; Ralls et al. 2000), and the captive environment itself will select for adaptations beneficial to captivity (Frankham 2008). In general, such adaptations do not favor survival and fecundity when organisms are released in to the wild (reviewed in Williams and Hoffman 2009).

Traditional management of the genetics of captive populations largely focuses on minimizing inbreeding and the loss of genetic variation, with occasional attention being given to ways of mitigating adaptation to captivity (see below). A cornerstone of this management is the equalization of founder representation in the population: this decreases selection (no variance in fitness) and slows the loss of genetic diversity. In practice, this is achieved using pedigree studbook information and ‘match-making’ sexual pairings that minimize the mean kinship between pairs. Despite the relatively beneficial population genetic outcome of such pairings, there has been little attention paid to potential genetic consequences of removing mate choice and sexual selection in captive breeding (but see Chargé et al. 2014; Quader 2005; Wedekind 2002). Sexual selection occurs through the competition for mates by one sex (usually males) and/or discriminating mate choice by the other (usually females). By allowing sexual selection in captive breeding, females would be able to choose among several males based on their secondary sexual characters. It has been suggested that sexual selection could improve purging of deleterious mutations and increase fitness in captivity because of mating with compatible individuals or individuals with ‘good genes’ (Whitlock and Agrawal 2009). In addition, the removal of mate choice in captivity will relax selection on female mate choice; potentially adding to the issues associated with genetic adaptation to captivity when individuals are released (e.g., females may become less adept at choosing the best males resulting in a general reduction in fitness). Behavioral biologists have promoted sexual selection as a potential tool for captive breeding management for over 15 years (e.g., Asa et al. 2011; Grahn et al. 1998; Quader 2005; Wedekind 2002). In 1998, Grahn et al. suggested that mate choice be given more consideration in conservation breeding programs, and in 2011, it was emphasized that the zoo community carefully considers mate choice implications for captive breeding (Asa et al. 2011). The zoo community is becoming increasingly interested in this discussion, especially when faced with reproductive failure of breeding pairs due to mate incompatibility or aggression which can lead to injury or death (Wielebnowski et al. 2002). More recently, the integration of sexual selection into captive breeding programs has been promoted through symposia that bring together researchers in the field of mate choice and zoo population managers (e.g., St. Louis Zoo, USA, 2010). Despite this, practical implementation of mate choice methods by the zoo community is very limited because they are ‘*interested in including mate choice but simply do not know how to go about it and/or unsure of the implications for genetic management’* (Asa et al. 2011). Thus, there is an urgent research need to assess the costs and benefits of allowing mate choice in breeding programs. However, the relative benefit of including management strategies that account for sexual selection in captive population evolution are uncertain and have received little attention.

In this paper, we briefly review current genetic management guidelines in captive breeding and the potential for conflict between these as a baseline for exploring how management techniques could be informed by sexual selection and mate choice theory, and what benefits these insights could bring to captive breeding and reintroduction biology.

## Current genetic management guidelines

**Figure 1:**
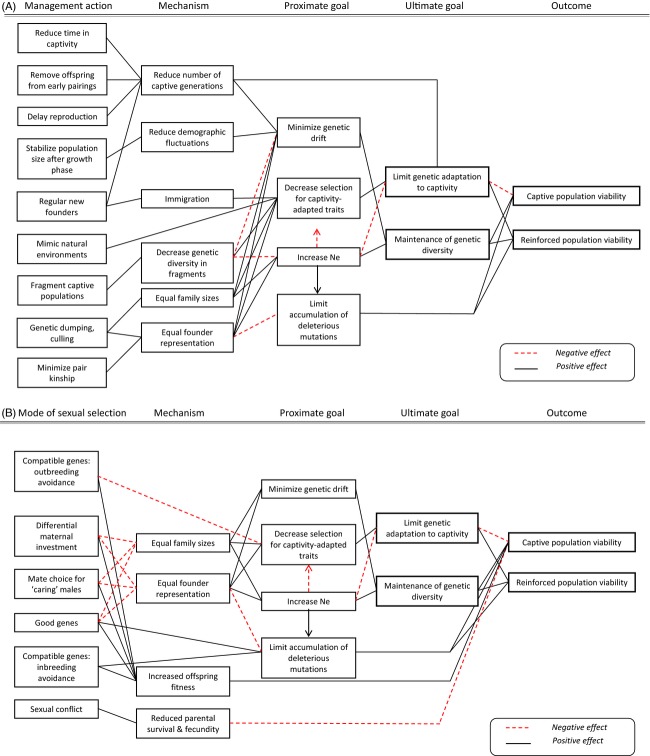
Interactions between management actions, goals, and outcome for the viability of captive and reinforced populations (A) and potential additional effects of sexual selection theories if female mate choice would be integrated to captive breeding programs (B). The direction of the linkages is from left to right unless otherwise specified by an arrowhead. Positive effects are indicated by a black line, negative effects by a red dashed line.

Breeding histories and conservation goals vary for each species in captivity, and although this suggests genetic management should be tailored to each population relative to its specific short- and long-term program goals (Earnhardt 1999; Fa et al. 2011), most captive breeding programs for conservation utilize similar guidelines aimed at minimizing the rate of loss of genetic variability and inbreeding depression (Frankham et al. 2000; Fraser 2008; Wang and Ryman 2001; Williams and Hoffman 2009; see Fig.1a).

### Maximizing N_e_/N ratio

The effective population size (N_e_) is generally smaller than the absolute population size (N), with N_e_ being the size of an idealized population with the same measure or rate of loss of some genetic quantity as that in the population under study (Allendorf and Luikart 2007). Because inbreeding depression and loss of heterozygosity are negatively related to N_e_ (Soulé 1980), one of the most important captive management aims for limiting loss of genetic diversity is to maximize N_e_ by equalizing family size, the sex ratio of breeders (Fa et al. 2011), and stabilizing population size after the initial population growth phase (Frankham 1995).

### Equalizing founder representation and minimizing inbreeding

Another important strategy to limit genetic change is to equalize the representation of each founder in the captive population by minimizing kinship of mated pairs (Ballou and Lacy 1995; Frankham et al. 2000; Lacy 2000) or by removing offspring from breeders with the highest mean kinship (e.g., culling or ‘genetic dumping’ in Earnhardt 1999). Mean kinship is high when individuals are over-represented in the population, and low when they represent rare founder genetic lines (Ballou and Lacy 1995; Grahn et al. 1998; Saura et al. 2008; Asa et al. 2011). When founder contributions are equal, this increases N_e_; thus reducing inbreeding and loss of genetic variation (Woodworth et al. 2002).

### Minimizing the rate of random genetic drift

Demographic fluctuations increase genetic drift in captive populations (Frankham 1995); thus population sizes are usually stabilized after the initial population growth phase (Fa et al. 2011). Another method to slow the rate of genetic drift over time is to increase the generation length by delaying reproduction of breeders or the removal of offspring from early pairings (Williams and Hoffman 2009).

### Limiting genetic adaptation to captivity

Organisms destined for subsequent reintroduction from captivity require genotypes suited to the reintroduction environment; however, genetic management for population viability in captivity does not take this into account. Indeed, there is increasing evidence that genotypes selected for under captive conditions are generally disadvantaged in natural environments (see (Frankham 2008; Williams and Hoffman 2009 for recent reviews). This has resulted in recent recommendations on how to manage genetic adaptation to captivity based on Frankham's (2008) equation, which positively relates the cumulative genetic change in reproductive fitness in captivity to selection, heritability, effective population size and, number of generations in captivity (see Box 1; Fig.1a). Based on this, four options for minimizing genetic adaption to captivity have been recommended; however, not all of these are practical and some are in conflict with recommendations designed to limit losses of genetic variability (for more discussion of conflicts see below). First is minimizing the number of generations in captivity, either by reducing the length of the captive period, using cryopreservation or increasing generation length (Frankham 2008). This is seen as the most efficient method available because of the exponential relationship between number of generations and adaptation (Box 1), but it is not often feasible. Second is minimizing selection by creating captive environments that mimic natural habitats and/or through breeding strategies that reduce selection: such as equalizing founder representation through managing kinship of mated pairs and equalizing family sizes (Allendorf 1993; Frankham 2008). Minimizing variability in reproductive success removes the between-family component of selection, potentially halving the rate of genetic adaptation to captivity (Frankham and Loebel 1992; Saura et al. 2008). Third is minimizing the effective population size. Because this is in direct conflict with recommendations to preserve genetic variability (see above), it has been suggested that both goals can be achieved through fragmenting the captive population in to smaller management units (Frankham 2008; Margan et al. 1998; see section below). Finally is managing the captive population as a ‘semi-closed’ system, and allowing the occasional recruitment of immigrants from wild populations to slow genetic adaptation (Frankham and Loebel 1992).

**Box 1 Factors determining genetic adaptation to captivity**

Frankham (2008) postulated that the cumulative genetic change in reproductive fitness in captivity over *t* generations (*GA*_*t*_) can be derived from the breeder's equation (Lynch & Walsh 1998) and is a function of the selection differential (*S*), heritability (*h*^*2*^), the effective population size (*N*_*e*_), and number of generations in captivity (*t*):
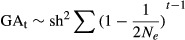
Thus, genetic adaptation to captivity will be positively related to the intensity of selection, genetic diversity, the effective population size, and number of generations in captivity (Frankham 2008; Williams and Hoffman 2009).

## Conflicts, trade-offs, and fitness losses

Several approaches for managing genetic adaptation to captivity are incidental to already established practices for managing genetic variability in captive populations (e.g., minimizing the number of generations, equalizing family sizes and founder representation, and allowing the occasional recruitment of wild genotypes). However, there is a direct conflict between the recommendation concerning ideal captive population size for minimizing genetic drift and inbreeding (large N_e_) and that for genetic adaptation to captivity (small N_e_; Fig.1a). This conflict between genetic goals is not a trivial concern as there is increasing evidence that adaptation to captivity in large populations can occur within very few generations (De Mestral and Herbinger 2013; Milot et al. 2013), resulting in serious fitness losses when organisms are released into the wild (reviewed in Williams and Hoffman 2009). Although much of the empirical evidence is still restricted to laboratory (e.g., Frankham and Loebel 1992; Lacy 2013) and commercial species (e.g., Laikre et al. 2010; Neff et al. 2011), it is well known that the reintroduction of organisms from captive breeding programs have lower fitness and lower probability of reintroduction success than those from the wild (Griffith et al. 1989; Wolf et al. 1996; Fischer and Lindenmayer 2000).

We see the potential for conflict in genetic management recommendations leading to three key decision making steps in captive breeding programs to limit fitness losses. First is assessing how long the captive population is expected to persist, how often and from where it may be reinforced, and if, when and how it may act as a source for reinforcing other populations (Lacy 2013). These program goals will largely determine how best to trade-off genetic variability against adaptation to captivity (step 2; see below), and how the needs of the captive population may be traded-off against the needs of reintroductions/wild supplementations (step 3). For example, if the captive population is being kept for reasons other than conservation reintroductions (e.g., public education), then adaptation to captivity effects can possibly be ignored or even promoted. There are fitness benefits to being well adapted to the local environment, so if populations will be permanently housed in captivity, behavioral and physiological adaptations suited to captivity may improve the fitness of captive animals (Woodworth et al. 2002) and thus the probability of long-term captive population persistence. The trade-off here being that organisms will change in some way from their wild counterparts, which may not be ideal if the purpose is to display or study ‘natural’ behaviors and morphologies, but they will change anyway; current genetic management in captivity is not a means of stopping genetic change, but simply slowing it (Lacy 2013).

Second, for those captive populations likely to be used for reintroductions or supplementations, how should effective population size in captivity be managed? Woodworth et al. (2002) show that fitness in captivity is expected to increase with increasing N_e_ because all genetic correlates with fitness operate in this direction. However, fitness in the wild after release shows a curvilinear pattern because of stronger adaptation to captivity with large N_e_, while inbreeding and mutational accumulation reduce fitness for small N_e_; thus, fitness is maximized in the wild after release from captive populations of a moderate size (Woodworth et al. 2002). Because of this relationship, it is now recommended that populations be managed in captivity through fragmentation (Margan et al. 1998; reviewed in Frankham 2008 and Williams and Hoffman 2009). This approach attempts to account for the opposing effects of N_e_ on fitness after release, whereby adaptation to captivity is reduced by fragmenting populations across institutions and allowing the small N_e_ to reduce genetic diversity at a local level (managing genetic adaptation to captivity), while retaining it at the metapopulation level (managing the loss of genetic diversity). Although the idea has theoretical and some empirical support, evidence from captive populations is extremely limited (Williams and Hoffman 2009).

Third, reintroducing captive animals to the wild is likely to involve a genetic trade off that is often not discussed, but one that may play a large role in reintroduction success (and future captive population viability)—that is, which animals should be released and which should remain in captivity? Earnhardt (1999) shows that the decision depends upon the relative value placed on the captive versus the reintroduced subpopulation. For example, one strategy (i.e., genetic dumping) promotes genetic diversity in the captive population at the expense of the reintroduced cohort; while minimizing kinship among released animals provides the greatest evolutionary potential for the release cohort, at the expense of the genetic health of the captive population. Thus, every reintroduction is a trade-off between the long-term persistence of the release and captive subpopulations and needs to be assessed on a case-by-case basis.

## Incorporating mate choice into captive management

Current captive breeding programs primarily focus on limiting the loss of genetic diversity through the careful management of sexual pairings (see above). The nonrandom access to breeding partners usually increases the among-individual variance in reproductive success with few individuals securing most of the fertilizations and therefore reducing effective population size and increasing inbreeding. For these reasons, captive breeding programs are mostly based on enforced monogamy. However, because of concerns that such management may increasingly limit population evolvability and fitness (e.g., for animals released back into the wild; Frankham 2008; Neff et al. 2011), it has been suggested that integrating sexual selection into the genetic management of captive populations, by allowing reproductive partners to express their mating preference, may help long-term population viability (Wedekind 2002; Asa et al. 2011; Pélabon et al. 2014). Female mate choice is a key component of sexual selection and is the area where most attention is currently being focused in captive management (Asa et al. 2011). Because of this, we will leave the potentially important male component of sexual selection (i.e., male–male competition and male mate choice) to future analysis; however we will discuss the importance of sexual conflict.

Sexual selection refers to the process of nonrandom mate choice that arises as a consequence of interindividual competition for sexual partners. This results in the evolution of sexually selected traits (e.g., mate choice preferences) that arise through direct benefits to females (e.g., increased fecundity or parental care, Andersson 1994) or indirect benefits to offspring (e.g., the inheritance of alleles that increase attractiveness, ‘sexy sons’, Fisher 1930) or viability (good genes, compatible genes, Candolin and Heuschele 2008). The link between mate choice and increased population viability can potentially be made for three mechanisms driving sexual selection: (i) direct benefits to females through increased female fecundity, (ii) increased genetic quality of offspring through additive genetic variation in fitness (good genes; e.g., Chargé et al. 2011), and (iii) increased genetic quality of offspring through nonadditive genetic variation (compatible genes) (Candolin and Heuschele 2008; see Box 2). Ideally, to ensure the long-term success of captive breeding and release programs, genetic diversity, and genetic quality have to both be maintained along generations in captivity. Any benefit of mate choice will depend on the specific program's goals. We explore the possible benefits and costs associated with incorporating mate choice below, as well as highlighting questions and assumptions we feel need to be addressed. Box 2 gives an overview of the main hypotheses that explain the costs and benefits of nonrandom mate choice in animals. Table1 and Fig.1 give an overview of the complex interactions between possible genetic benefits and risks associated with the integration of the main sexual selection theories (Fig.1b) into the current genetic management of captive populations (Fig.1a).

**Box 2 Fitness benefits associated with females mate choice**

The utility of male attributes selected via female mate choice is species-specific and likely to include one or a combination of the following:

**Direct benefits:** Females can attain direct fitness benefits from choosing mates that improve their own fecundity; such as the male's ability to nest build, rear offspring, courtship feed, or provide other valuable resources within the territory (Norris 1990; Møller 1994; Brown 1997). Female choice may also work to limit negative fitness consequences of pairing by avoiding unhealthy males, sexually transmitted diseases, or male infertility (e.g., by selecting feather brightness in birds;.(Hamilton and Zuk 1982; Kokko et al. 2002; Matthews et al. 1997; Pitcher and Evans 2001).

**Differential maternal investment:** Females may adjust their investment in offspring depending on male attractiveness. In mallards (*Anas platyrhynchos*), females laid more eggs when mated with preferred males (Cunningham and Russell 2000). In the Houbara bustard (*Chlamydotis undulata*), artificially inseminated females that were visually stimulated by attractive males had better hatching success and increased chick growth compared to those stimulated with less attractive males (Loyau and Lacroix 2010).

**Good genes:** Females may choose male phenotypes indicative of ‘good genes’, which improve the fitness of their progeny (Andersson 1994; Møller 1994; Neff and Pitcher 2005). The parasite-mediated sexual selection theory predicts that these good genes play a crucial role in parasite resistance (Hamilton and Zuk 1982), with offspring being more resistant to local pathogens, and thus conferring higher fitness (Buchholz 1995; Kirkpatrick and Ryan 1991; Penn and Potts 1999). There is increasing support for the degree of male ornamentation (and female preference for it) being correlated with genetic quality (see ‘the handicap principle’; Zahavi 1975).

**Compatible genes:**

**Inbreeding avoidance:** Females may choose males based on the degree of relatedness to limit inbreeding depression (Kempenaers 2007). In guppies (*Poecilia reticulate*), females prefer to mate with males newly introduced or with rare phenotypes (Hugues 1991); in chickens, *Gallus gallus*, females hold less sperm after insemination by one of their brothers (Pizzari et al. 2004).

**Outbreeding avoidance:** Females may avoid outbreeding in order to maintain local adaptations, to select males to optimize the degree of relatedness, or simply to increase the representation of genes identical by descent (Höglund et al. 2002; Puurtinen 2011). For instance, house sparrow (*Passer domesticus*) males failed to form breeding pairs with females too dissimilar at major histocompatibility complex (MHC) loci (Bonneaud et al. 2006). Peron's tree frog (*Litoria peronei*) males that were genetically similar to the female achieved higher siring success than less genetically similar males (Sherman et al. 2008). In the three-spines sticklebacks, female seems to be able to ‘count’ the number of MHC alleles in the sexual partner and choose males that share an optimum number of alleles with them (Aeschlimann et al. 2003).

**Heterozygote advantage in offspring:** Females may also seek to maximize heterozygosity in the offspring at key loci or at many loci (Brown 1997). For instance, in the domestic sheep, homozygous ewes inheriting mutant alleles from both parents have lower fecundity compare to heterozygous individuals for the same loci expressing increased ovulation rate (Gemmell and Slate 2006). Females may also try to maximize the offspring heterozygosity at key loci such as at MHC genes (reviewed in Penn 2002). In mice (and in humans), females prefer to mate with males carrying dissimilar MHC alleles than their own (Wedekind and Furi 1997; Penn and Potts 1998) which may enhance offspring immunocompetence. Although there is little evidence from tests of single parasites to support this hypothesis, MHC-heterozygous offspring may be resistant to multiple parasites (Penn and Potts 1999 and references within).

**Sexy sons:** Females may express a preference for heritable attractiveness in males, regardless of the utility of the trait. This may occur if the genes for the female preference become associated in linkage disequilibrium with genes for the trait underlying males attractiveness; females will select males that also carry the genes for the female preference of that male trait. This produces a positive feedback ‘runaway’ loop that is assumed to lead to the extravagance of male traits until the costs of such secondary sexual traits in terms of survival exceed the benefits in term of reproductive success (Fisher 1930; Weatherhead and Robertson 1979). Empirical evidence comes from studies on fruit flies and European starlings (Gwinner and Schwabl 2005; Taylor et al. 2007).

**Table 1:** Potential benefits (B) and risks (R) from integrating theories of female mate choice into captive breeding programs for the viability of the captive population and that of any cohorts released into the wild. See Box 2 for a summary of each theory. When no benefit or risk was obvious, we indicate it by ‘?’; however, it suggests that more research is needed rather than implies that no risks can be safely assumed.

Theory	Impact on captive population	Impact on released cohort
Direct benefits/Maternal investment		
B	Increase female fecundity, lifespan, and offspring viability	Maintain males secondary sexual traits
	Select healthier males that afford expressing strong parental effort	
R	Decrease lifespan reproductive success if trade-off with parental effort	Select males adapted to captivity
Good genes		
B	Purge deleterious alleles	Select resistant individuals (e.g. if similar pathogens in the wild and in captivity)
R	Loss of genetic variance	Loss of genetic variance
	Decrease female fitness in case of sexual conflict	Select males adapted to captivity
	Decrease some fitness traits in males (e.g. if trade-off between immunity, reproduction, and lifespan)	Decrease female fitness in case of sexual conflict
Compatible genes		
Inbreeding avoidance		
B	Minimize inbreeding depression	Minimize inbreeding depression
R	Misled mate choice between kinship and familiarity	Loss of local adaptationMisled mate choice between kinship and familiarity
Outbreeding avoidance/‘(k)inbreeding selection		
B	?	Maintain local adaptation
R	Increase risks of inbreeding depression	Increase risks of inbreeding depression
Maximizing heterozygosity in the offspring		
B	Minimize inbreeding depression	Minimize inbreeding depression
	Improve offspring viability	
	(heterozygous advantage)	Improve offspring viability (heterozygous advantage)
R	?	?
Sexy sons		
B	Maintain male ornamentation and female preferences	Maintain male ornamentation and female preferences
R	Decrease female fitness in case of sexual conflict	Select males adapted to captivity
		Decrease female fitness in case of sexual conflict

### Direct benefits and differential maternal investment

Equally relevant for guiding the choice of enforcing monogamy in captive breeding is the observation that multiply mated females usually adjust the investment they make into offspring, affecting progeny quality and survival. Multiple lines of evidence show that females adjust their investment in offspring depending on the male they are mated to (Gil 1999); for example, when mated to preferred males (i) female mallards lay more eggs (Cunningham and Russell 2000), (ii) female house mice produce larger litter sizes (Drickamer et al. 2000), and (iii) female birds, insects, and crustacea deposit more testosterone in their eggs (Gil 1999; Kotiaho et al. 2003; Galeotti et al. 2006; Loyau et al. 2007). More recently, differential maternal investment has been investigated in supportive captive breeding of the endangered Houbara bustard. Artificially inseminated females visually stimulated by attractive males increased their hatching success as well as the allocation of androgens in their eggs and increased growth rate in chicks (Loyau and Lacroix 2010). Here, it was emphasized that using artificial insemination for species conservation without appropriate stimulation of the breeding females may lower their breeding performance with negative impact on the population viability. Thus, maximizing parental effort by allowing free mate choice in captive-bred populations might increase offspring quality and help in the long-term viability of captive and reinforced wild populations (Asa et al. 2011). However, while this expectation seems reasonable, it is unlikely to be this straightforward (Kokko and Brooks 2003). In a recent review of current progress in implementing mate choice in captive breeding programs (Asa et al. 2011), the zoo community's initial steps are primarily focusing on the direct benefits of female mate choice to improve the probability of successful mating in valuable animals. While there is a general perception that giving animals choice should improve female fecundity (and consequently improve population persistence), there are a number of issues that need to be clarified from a captive breeding perspective. First is the general problem with female choice increasing the variance in reproductive success, thereby decreasing effective population size and increasing any imbalance in founder representation (Wedekind 2002). Thus, including mate choice in breeding management appears to directly conflict with current management goals that aim to minimize the loss of genetic variation and adaptation to captivity (Asa et al. 2011): adding an additional level of complexity in determining the best breeding strategy for captive populations (see above). Second is the idea that females in captivity are able to make accurate choices about male quality. Managers need to be clear on whether they are providing real choice for females to find the best mates or simply providing a ‘simulation’ of natural breeding to ‘trick’ females into increasing their reproductive investment accordingly. If we want females to make informed mate choice decisions, this makes a very strong assumption that male quality under captive conditions can be differentiated by females, even when limiting resources have been provided for. For example, if male coloration in the wild is a cue for health, territory quality, or foraging ability (e.g., Wolfenbarger 1999; Saks et al. 2003; Karino et al. 2005), how is it expressed under captive conditions where veterinary care is ongoing, food is provided *ad libitum*, and housing is standardized? Thus, the expected fecundity benefits in captivity may be much smaller (or even absent) compared to studies from wild populations where female choice is correlated with a limiting resource being provided by males. Third is the possibility that reproduction and survival (or current versus future reproduction) are traded-off against each other (Saino et al. 1999). Thus, it is possible that by promoting current reproductive output via direct benefits, future reproductive potential may be compromised; however, these effects are predicted in wild populations, and it is generally unknown how such relationships are affected by captive environments where key resources may not be limiting.

### Benefits of sexual selection for population fitness and adaptation rate

Sexual selection can be a powerful force contributing to purge deleterious mutations from the genome, and theoretical work has shown that this can produce a net benefit that can improve population mean fitness and the rate of adaptation (Agrawal 2001; Siller 2001; Lorch et al. 2003; Whitlock and Agrawal 2009). Testing the benefit of sexual selection for population mean fitness, and the rate of adaptation has been achieved through experimental evolution approaches where females were either forced to mate under a monogamous regime or were allowed to mate with several males. For obvious reasons linked to generation time and laboratory facilities, this approach has mostly involved insects and other invertebrates, with a couple of notable exceptions involving guppies (*Poecilia reticulata*) and house mice (*Mus domesticus*; see examples below and Holman and Kokko 2013 for a recent overview of the topic).

In an elegant experiment, Almbro and Simmons (2014) exposed dung beetles (*Onthophagus taurus*) to a mutagenic treatment with ionizing radiation and then selected beetles under either enforced monogamy or sexual selection. After only two generations of sexual selection regime, the expression of male strength, a sexually selected trait, of irradiated beetles was almost twice as large as for the monogamous lines, and almost recovered the values of nonirradiated control individuals. In guppies, Pélabon et al. (2014) conducted an experimental evolution study where 19 populations of guppies were exposed to an enforced monogamous or a polygamous mating system for nine generations. Offspring size decreased across generations in both regimes, but the decrease was more pronounced in the enforced monogamy treatment. Therefore, despite being held in a benign (captive) environment for only nine generations, preventing mate choice and sexual selection resulted in the reduction in the expression of a trait that is potentially correlated with fitness (both sexual and nonsexual) in the wild. In the only mammalian species where the effect of mating system has been investigated, the house mouse, females that were free to mate with preferred mates produced (i) more litters, (ii) socially dominant sons, (iii) offspring with a better survival compared to females forced to mate with nonpreferred males (Drickamer et al. 2000). In addition to this, an experimental evolution approach where house mice were either polygamously or monogamously mated during 14 generations showed that offspring viability was improved when they were sired by males that had experienced the polygamous selection regime (Firman and Simmons 2012). Therefore, in the only study where divergent selection lines for mating system have been used in a mammal, sexual selection appears to confer a long-term fitness benefits to males and females, suggesting concordant effect on sexual and nonsexual traits.

Ultimately, if sexual selection produces a net benefit on population mean fitness, this should reduce the population extinction risk. Jarzebowska and Radwan (2009) used small populations (five males and five females) of the bulb mites (*Rhizoglyphus robini*) facing either enforced monogamy or sexual selection and looked at the extinction probability of each line. They found that 49% of the lines in the monogamy treatment went extinct versus 27% in the sexual selection group. In a similar experiment using the same species, Plesnar-Bielak et al. (2012) showed that the extinction probability of lines selected under enforced monogamy or sexual selection markedly differed when exposed to a harsh environment (a temperature stress): 100% of monogamous lines went extinct when reared at high temperature versus 0% for lines experiencing sexual selection.

### Costly sexual traits and sexual conflict

Despite some studies providing supportive evidence that sexual selection promotes population mean fitness, this is not always the case as several examples show sexual selection does not purge deleterious mutation nor improve population fitness (in *Drosophila melanogaster*, Arbuthnott and Rundle 2012; Hollis and Houle 2011) or on the rate of adaptation to a novel environment (in the yeast, *Saccharomyces cerevisiae,* Reding et al. 2013). Moreover, sexual selection can favor the evolution of traits that have fitness costs and are instead associated with mating success (sexy sons, Fisher 1930; signal honesty, Zahavi 1975 or sexually antagonistic coevolution, Holland and Rice 1998). The Fisher–Zahavi traits evolve so that the benefits to the male in terms of mating success from female preferences are balanced by the costs of the traits. Because there are no population benefits involved, sexual selection primarily driven by these processes might be a burden when conditions change. This occurs because sexual selection is expected to exert its strongest negative effects on population viability under rapidly changing conditions when there is not enough time available for the costs of sexual traits to be adjusted to the new conditions (Candolin and Heuschele 2008). This is particularly relevant to understanding the possible role of sexual selection on the adaptation of captive populations to a novel environment, but the effect for most populations is currently unknown.

Sexual selection through antagonistic selection is a widespread phenomenon (Cox and Calsbeek 2009) that has been well documented and its associated theoretical framework intensively tackled (reviewed in Bonduriansky and Chenoweth 2009; Cox and Calsbeek 2009; Van Doorn 2009). Two main forms of sexual conflict can be distinguished: the antagonistic interactions between the sexes (i.e., interlocus sexual conflict) and the genetic trade-offs for fitness between males and females (i.e., intralocus sexual conflict). Interlocus sexual conflict occurs when traits coded by alleles at different loci evolved so that it enhances the reproductive success in individuals from one sex at the cost of the fitness of their mating partners (Chapman et al. 2003; Bonduriansky and Chenoweth 2009). Behavioral examples include sexual coercion, mate guarding or mating plug, physical or physiological harassment of the partner, evasion of parental care, and resistance against mating (Chapman et al. 2003; Van Doorn 2009 and references within). By contrast, intralocus sexual conflict arises when the same set of fitness-related loci between sexes is subject to opposing selection pressures, preventing males and females from reaching their optima independently (Lande 1980; Chippindale et al. 2001). For instance, some secondary sexual traits in males improve male–male competition and mating success but are costly to produce for females, like horn phenotype in the Soay sheep, *Ovis aries* (Robinson et al. 2006) and red bill color in zebra finches, *Taeniopygia guttata* (Price and Burley 1994). In red deer, *Cervus elaphus*, selection favors males that carry low breeding values for female fitness resulting in the situation where males with relatively high fitness sired daughters with relatively low fitness (Foerster et al. 2007). Intralocus sexual conflict is controversial because such conflict is thought to be resolvable through the evolution of sex-specific gene expression, sex-linkage, and sexual dimorphism, enabling each sex to reach its adaptive optima (Bonduriansky and Chenoweth 2009; Cox and Calsbeek 2009; Stewart et al. 2010). But recent studies have shown that the conflict was not so easily resolved (Harano et al. 2010; Poissant et al. 2010; Tarka et al. 2014).

Such sexual conflicts may be relevant to population persistence, population genetics, and adaptation. When sexual conflict favors males, female fecundity is often reduced which may affect in turn population demography, mean population fitness, and increase extinction risks (Kokko and Brooks 2003; Rice et al. 2006; Morrow et al. 2008; Bonduriansky and Chenoweth 2009). It is thus important to account for sexual conflicts in the captive breeding programs to predict long-term outcomes of sexual selection on captive and reinforced population viability. For instance, in the lizard *Lacerta vivipara,* male sexual behavior is harmful and male-skewed sex ratios can threaten population persistence (Le Gaillard et al. 1998; see also Low 2005).

Because inter- and intralocus conflict have different genetic consequences, it is important to distinguish the evolutionary outcomes from both strategies. Although evolutionary outcomes of sexual conflicts are not yet fully understood, we briefly synthesize current knowledge. Interlocus sexual conflict generates coevolutionary arms races which have been thought to accelerate evolution of traits, particularly the antagonistic evolution of reproductive traits (Van Doorn 2009; Arbuthnott and Rundle 2012); this opposes the goal of captive breeding programs to maintain genetic diversity and prevent (response to) selection. Interlocus sexual conflict resulting in direct harm to females could be compensated by indirect genetic benefits (good genes or sexy sons, Cox and Calsbeek 2009). However, several empirical studies failed to show that costs related to sexual conflict were counterbalanced by good genes (Stewart et al. 2008), sexy sons (Rice et al. 2006), or compatible genes (Garner et al. 2010).

The evolutionary importance of intralocus sexual conflict is still debated (Chapman et al. 2003; Cox and Calsbeek 2009), with current evidence suggesting that when intralocus sexual conflict occurs across multiple loci, the so-called tug-of-war can neutralize benefits from sexual selection (Cox and Calsbeek 2009 and references within) and reduce population mean fitness (Bonduriansky and Chenoweth 2009). Paradoxically, theory also suggests the potential role of intralocus sexual conflict in maintaining genetic variation, although this idea has received little attention so far (Foerster et al. 2007). Antagonistic selection may maintain substantial levels of genetic variation in life history traits despite the directional selection to which they are subject (Kruuk et al. 2000); data from red deer natural populations show that sexually antagonistic selection could maintain heritable genetic variance in reproductive traits and fitness variation. Similarly in *Drosophila melanogaster*, gender-specific selection on loci expressed in both sexes may contribute to the maintenance of high levels of genetic variance for fitness within each sex (Chippindale et al. 2001). Sexual conflict could thus maintain genetic variation for fitness despite strong selection (Foerster et al. 2007). This genetic outcome may be of particular interest for the management of captive populations, but a detailed understanding of the strength of intralocus sexual conflict and its contribution to the maintenance of genetic variation will clearly require careful consideration (Foerster et al. 2007; Bonduriansky and Chenoweth 2009; Cox and Calsbeek 2009).

The net benefit of allowing sexual selection to operate likely depends on the relative importance of costs induced by sexual conflicts and benefits induced by the purging of mutational load. In some cases, environmental condition and population history can strongly modulate the net benefit of sexual selection. For instance, if populations are exposed to the arrival of newly maladapted alleles, the benefit of purging these alleles might outweigh the potential cost due to sexual conflicts. Long et al. (2012) have recently tested this idea using experimental populations of *Drosophila melanogaster* that were either well adapted to their environment (cadmium-adapted populations), either pushed away from their adaptive peak by the income of migrant, maladapted, alleles. For each of these populations, they identified sexually successful and nonsuccessful males and used them to sire offspring. In agreement with the predictions, they found that sexually successful males sired unfit daughters in well-adapted populations, which corroborate the finding that sexual conflict produces a mismatch between sexual and nonsexual fitness in this species. However, sexually successful males sired fitter daughters in the populations where adaptation was prevented by the income of migrant alleles. This suggests that in unstable populations, the net benefit of purging deleterious alleles outweighs the cost of sexual conflicts. These results are mirrored by those reported by another recent study where the outcome of exposure to a regime of enforced monogamy versus polyandry depends on environmental quality (Grazer et al. 2014). Flour beetles (*Triboleum castaneum*) were maintained for 39 generation either under enforced monogamy or polyandry. Beetles from these selection lines were exposed to a poor or a good environment in terms of food quality. Reproductive success of pairs formed by males from the sexual selection lines and females from the enforced monogamy was low when reared in the good environment, again suggesting that sexual conflict incurs cost. However, when sexually selected males were mated with enforced monogamous females in the poor-quality environment, their reproductive success was improved suggesting that the benefits of sexual section outweighed the cost of sexual conflicts under stressful conditions. Despite the evidence of a net benefit of female choice to population viability from many of these studies, and hence suggesting that captive population management would benefit by incorporating female choice, these ‘benefits’ have generally not been considered within the complex framework of interactions and conflicting goals for long-term population persistence (e.g., Fig.1). Thus, we encourage caution before female choice measures are adopted in captive breeding programs (see below).

## Conclusions

To date, the main genetic focus of captive breeding programs has been on preserving genetic diversity, while genetic integrity is often neglected because of difficulty in measuring progress and conflicts with other genetic guidelines (on the basis of N_e_). One means of preserving genetic integrity is incorporating female choice for male traits in captive breeding management. Based on current limited theoretical and empirical evidence, it appears that some mechanisms for mate choice may be safer to exploit than others. On the safer side are female preferences for compatible genes, general heterozygosity or allelic diversity at specific locus (e.g., major histocompatibility complex (MHC) genes), and differential maternal investment based on male's attractiveness. At the riskier end of the spectrum is selection for good genes in the presence of sexual conflict, as this could favor adaptation to captivity in males while decreasing female fitness by creating unbalanced selection pressures with sexual selection on males, while natural selection is lifted on females. One possibility we have not explored in our review is sexual selection acting on females through male choice and female–female competition; if and where this occurs, it could help balancing selection on both sexes and potentially obtain better results in terms of fitness for both. Another area still unexplored is the potential for integrating male–male competition; however, the risks of favoring males best adapted to captivity would likely be the same as in the good genes hypothesis.

Although there has been increasing attention focused on mate choice as a potential way of improving captive population management, its impact on genetic variability and adaptation to captivity is complex (Fig.1). Incorporating mate choice into captive breeding recommendations presents a huge challenge, both in terms of the logistics of offering mate choice in captive settings and in implementing choice in a way that augments rather than hinders population management goals (Asa et al. 2011; see Fig.1). Despite this, progress is possible, and a first step is identifying the key questions that need to be asked before considering implementing mate choice into a breeding program. First is assessing how long the captive population is expected to persist, how often and from where it may be reinforced, and if, when and how it may act as a source for reinforcing other populations (Lacy 2013). This should be a first step in any decision regarding the genetic management of captive populations because it determines how genetic adaptation to captivity needs to be considered, especially if mate choice accelerates adaptation to captivity. Second is identifying the mechanism (or sexual selection theory) driving mate choice in the system of interest. Is it likely that mate choice is linked to improved population persistence, and if so, are the expected benefits likely to be via improved fecundity of breeding females or the genetics of offspring? Also, is it reasonable to expect that phenotypic traits in males that females select on are still valid cues for genetic quality in captivity? Third is identifying whether sexual conflicts exist in the mating system. Fourth is considering the potential for conflicts with other genetic management goals. Because mate choice increases variation in mating success, this will generally reduce effective population size and erode genetic diversity in the captive population; thus, the benefits of incorporating mate choice will need to be balanced against any costs.

Currently, we need specific questions to be asked that link directly to the needs of captive management and then specific studies implemented (both empirical and via simulation studies) to look at specific management approaches, such has been successfully achieved in identifying ways to manage genetic adaptation to captivity through population fragmentation (Margan et al. 1998; Frankham 2008). It is only then that we will begin to seriously contribute to the genetic health of captive populations and the success of reintroductions. Thus, the goal of this review has not been to provide definitive answers and recommendations on the benefits (and costs) of mate choice and sexual selection in the management of captive populations, but rather to highlight the complexity of the relationships between mate choice, population fitness, and the current genetic goals of maintaining small populations in captivity. From this, we hope to encourage clear goal-oriented research and critical thinking into the role of mate choice and sexual selection in an area where its application and study are currently in its infancy.
